# Temperature and Aging Effects on the Rheological Properties and Performance of Geopolymer-Modified Asphalt Binder and Mixtures

**DOI:** 10.3390/ma16031012

**Published:** 2023-01-22

**Authors:** Abdulrahman Hamid, Hassan Baaj, Mohab El-Hakim

**Affiliations:** 1 Department of Civil and Environmental Engineering, University of Waterloo, Waterloo, ON N2L 3G1, Canada; 2 Department of Civil and Environmental Engineering, Manhattan College, Bronx, NY 10471, USA

**Keywords:** rheology, geopolymer, SBS, fatigue, moisture damage, fracture energy

## Abstract

Using geopolymer as a modifier for asphalt binders and mixtures gained momentum for investigation in recent decades. Limited research investigations attempted to link the effect of temperature and traffic loading on the rheological properties and performance of geopolymer-modified asphalt binders. The primary objective of this study is to evaluate the effect of fly ash-based geopolymer (GF), styrene-butadiene-styrene (SBS), and the combination of GF with SBS on the rheological properties and performance of asphalt binders at low and intermediate temperatures. The rheological properties and performance of neat and modified asphalt binders (4%GF, 8%GF,12%GF, 2%SBS, and hybrid (2%SBS+8%GF)) were evaluated utilizing dynamic shear rheometer (DSR) and bending beam rheometer (BBR) devices. To evaluate the fatigue resistance of asphalt binders, the linear amplitude sweep (LAS) and viscoelastic continuum damage (VECD) models were applied. The dynamic or complex modulus and moisture damage resistance were measured to investigate the influence of modifiers on the performance of asphalt mixtures. The findings demonstrated that for both unaged and RTFO-aged asphalt binders, additives reduced the temperature sensitivity of both G′ and G″. When the binders were exposed to long-term aging using a pressure aging vessel (PAV), it was noticed that the 8%GF binder became more susceptible to temperature changes. The 2%SBS binder had the lowest creep stiffness compared with the neat and other modifiers, while the hybrid binder exhibited the highest resistance to fatigue distress at different temperatures compared with the other binders. The modified asphalt mixes (8%GF, 2%SBS, and hybrid) achieved the maximum tensile strength ($S_{t}$) compared with the neat asphalt binder, with an increase of more than 80%. The $S_{t}$ increased from 580.4 kPa to 740.4 kPa, 884.8 kPa, and 917.4 kPa by utilising the 8%GF, 2%SBS, and hybrid binders, respectively. Furthermore, the modified asphalt mixture exhibited more ability to resist cracking, attaining the highest fracture energy in dry and freeze-thaw conditions.

## 1. Introduction

The performance of asphalt mixtures depends on numerous parameters, including the rheological properties of the asphalt binder, volumetric characteristics of the asphalt mixture, and external influences such as the traffic spectrum and weather. Asphalt binder is a great adhesive material used in roadway paving, but because of its complex behavior, it is a challenging material to evaluate and characterize. An asphalt binder’s behavior is characterized by viscoelastic properties. Based on the loading and temperature, it has both elastic and viscous properties. Asphalt binder is exposed to a wide variety of loading spectra and temperatures. A binder’s behavior leans toward soft viscous material in hot weather and brittle behavior in cold conditions. One of the primary reasons for pavement distress is the overload traffic spectrum, which causes significant stresses within the pavement layers. These distresses decrease the pavement’s serviceability and increase the maintenance cost. There are numerous methods for reducing pavement distress, including a balanced mix design and the use of modifiers [1].

The major motivation for modifying asphalt binders with various types of additives is to produce softer mixtures at low temperatures and prevent cracking. This leads to enhancing the mixture’s stability and strength, consequently increasing the rutting and fatigue resistance. Enhancements in a mixture’s resistance to rutting and fatigue could be utilized to decrease the pavement’s structural thickness. Bahia et al. (1997) [2] classified asphalt modifiers into groups, such as anti-stripping agents, hydrocarbons, crumb rubber, fillers, polymers, and fibers, according to their composition and effects. These additives have a wide range of physical and chemical properties that would impact the rheology and performance of asphalt mixtures in various aspects. The most frequent type of asphalt binder modifier investigated in various research studies is polymers. The polymer’s nature and content strongly enhance the asphalt binder’s rheological properties, increasing the stiffness and decreasing the phase angle (δ) [3] and temperature susceptibility [4].

Geopolymer is an inorganic polymer that incorporates the properties of polymers and cement by combining alumina and silica using an alkaline solution with a high concentration. Historically, the Cheops Pyramid in Gaza was built using formed in situ blocks made of alkali-activated aluminosilicate minerals [5]. Geopolymer belongs to the alkali-activated cementitious materials group, which has a low calcium concentration [6]. Geopolymers are materials that are favorable to the environment since they emit little CO${}_{2}$ during production and can reduce byproducts and waste materials. Additionally, geopolymer has shown that it can enhance mechanical properties, increase fire resistance, and decrease energy use and greenhouse gas emissions [7,8,9]. Geopolymer materials are employed in the treatment of toxic waste due to their capability to absorb harmful chemical pollutants [10]. Davidovits (1989) [11] reported that geopolymers such as zeolites and feldspathoids can adsorb harmful chemical contaminants. These operate as a binder to turn semi-solid waste into an adhesive solid and bind toxic elemental waste inside the geopolymer framework. The geopolymer matrix’s three-dimensional framework retains hazardous components in waste materials blended with geopolymer compounds. Zain et al. (2017) [12] concluded that the GF material performed best in terms of its aluminosilicate content and is currently a great method in environmental protection applications. The reaction of the alkaline solution (with a high concentration) with the aluminosilicate source resulted in the formation of the geopolymer. The chemical reactions that occur during the geopolymerization process are described by Equations (1) and (2) [13]:

n(Si${}_{2}$O${}_{5}$,Al${}_{2}$O${}_{2}$)+2nSiO${}_{2}$+4nH${}_{2}$O+NaOH

(1) $$\to Na^{+}+n{\left(OH\right)}_{3}-Si-O-Al^{-}{\left(OH\right)}_{2}-O-Si^{-}{\left(OH\right)}_{3}$$

n(OH)${}_{3}$-Si-O-Al${}^{-}$(OH)${}_{2}$-O-Si-(OH)${}_{3}$+NaOH

(2) $$\to Na^{+}-\left(Si-O-Al^{-}-O-Si-O-\right)+4nH_{2}O$$

Meanwhile, the use of geopolymers in various applications, including pavement construction, soil stabilization, and geopolymer concrete and mortar, has received a great deal of attention in recent decades. Geopolymers have been commonly employed as an environmentally friendly additive to cementous construction materials over the last few decades due to their ability to reduce CO${}_{2}$ emissions [14]. One ton of kaolin-based geopolymer generates 0.180 tons of CO${}_{2}$, which is six times less than Portland cement production. Additionally, GF production generates equal to nine times less CO${}_{2}$ than Portland cement manufacturing [10]. For instance, it is mixed with concrete for casting structural elements [8,15]. Nmiri et al. (2017) [16] studied the possibility of using kaolinitic clay material as an aluminosilicate source to produce geopolymer concrete. The clay was heated to a temperature between 550 and 1100 °C. NaOH and KOH solutions at concentrations of 5, 8, 10, 13, 15, or 18 molar were used to activate the calcined clay. The mineral and chemical composition of clay and geopolymer was characterized by utilizing X-ray diffraction, infrared spectroscopy, and thermal analysis. The compressive strength, porosity, and water absorption were utilized to characterize the physicochemical characteristics of the samples. According to the findings, a temperature of around 700 °C and an alkaline concentration of 13 molar are necessary to develop a metakaolin with the maximum degree of reactivity, whereby the various physical characteristics reach their optimal levels. However, the compressive strength, porosity, and water absorption started declining at higher temperatures. Hadi et al. (2017) [17] produced geopolymer by using slag as an aluminosilicate source and NaOH and Na${}_{2}$SiO${}_{3}$ as alkaline activators. The Taguchi method was utilized to design the optimum mix proportions. The effects of the concentration of NaOH, sodium silicate (Na${}_{2}$SiO${}_{3}$), alkaline-activator-to-binder-content (Al/Bi), and alkaline-activator-to-NaOH ratio were studied. The findings were that the specimens with the maximum seven-day compressive strength (60.4 MPa) had a binder content of 450 kg/m${}^{3}$, an Al/Bi ratio of 0.35, a Na${}_{2}$SiO${}_{3}$/NaOH ratio of 2.5, and a NaOH concentration of 14 molar. On the other hand, fly ash (FA), metakaolin (MK), and silica fume (SF) were utilized to enhance the setting time of geopolymer concrete by partial replacements for slag in various amounts.

Additionally, geopolymer was used to enhance soil characteristics. Geopolymer has a significant influence on clayed soil characteristics, and it can also be used as an effective alternative binder to ordinary Portland cement (OPC) in soil stabilization [18,19]. Abdullah et al. (2017) [19] investigated the potential usage of slag and FA geopolymers for stabilizing clay soil. It was noted that the replacement of FA using slag in a geopolymer-clay mixture induces a significant increase in soil strength. Ghadir and Ranjbar (2018) [18] stabilized clay soil using geopolymer and OPC and compared the mechanical performance, where the impact of the curing time and conditions, volcanic ash/clay ratio, and alkali activator were investigated. It was found that the alkali activator had a substantial impact on the compressive strength of the geopolymer-treated soil, and the soil stabilized by OPC had less ductility compared with the geopolymer-treated soil. Table 1 summarizes the types of geopolymer that were utilized to stabilize different types of soil. Phummiphan et al. (2018) [20] used high-calcium GF to stabilize silty clay sand, while slag was utilized as a replacement material in the soil. Three factors—slag content, curing time, and Na${}_{2}$SiO${}_{3}$/NaOH ratio–were utilized to investigate the influence of stabilizers on the soil properties. The results indicated that the soaked seven-day UCS of the stabilized soil with different ratios of Na${}_{2}$SiO${}_{3}$/NaOH attained the strength for both high- and low-volume roads, and 10% slag is suggested at high Na${}_{2}$SiO${}_{3}$/NaOH ratios (>80:20). Sore et al. (2018) [21] studied the feasibility of using geopolymer to stabilize compressed earth blocks (CEBs). Geopolymer was formed using metakaolin as an aluminosilicate source and NaOH with a concentration of 12 molar as an alkaline activator. Different percentages of geopolymer were utilized to stabilize the CEBs. The physical, mechanical, and thermal characteristics of CEBs with geopolymers were compared between CEBs and CEBs with 8% Portland cement. The results indicated that using geopolymer with CEBs greatly enhanced their mechanical performance and developed thermal characteristics which were quite close to those of CEBs without geopolymer, while stabilizing the CEBs with 15% geopolymer achieved properties similar to Portland cement-stabilized CEBs, particularly in terms of water resilience.

Recently, geopolymer has been used in pavement construction. Mohammadinia et al. (2016) [30] investigated how recycled construction and demolition (CD) materials including geopolymers behaved. The geopolymers were developed by combining various percentages of FA and slag (S) (4% FA, 2% FA+ 2% S, or 4% S) as an aluminosilicate source and NaOH and Na${}_{2}$SiO${}_{3}$ as alkaline activators. The geotechnical engineering and strength parameters of these materials were tested to determine their performance for pavement base and subbase applications. For unconfined compression and repeated load triaxial testing, the effect of the curing time on the strength of CD materials was investigated. The findings showed that using geopolymer enhanced the resilient modulus and compressive strength, which were increased. The compressive strength of slag-based geopolymer stabilization was higher than that of GF stabilization, while using geopolymer as a RAP stabilizer is a realistic and long-term solution for future pavement bases and subbases. Hoy et al. (2016) [31] conducted an environmental evaluation of GF-stabilized RAP. High-calcium FA is used as a source for aluminosilicate, with Na${}_{2}$SiO${}_{3}$ and NaOH as alkaline activators. XRD and SEM techniques were used to investigate microstructural development. The unconfined compressive strength of RAP-FA blends and RAP-GF were compared to the requirements of road authorities. The toxicity characteristic leaching procedure was utilized to determine the heavy metals’ leachability and compare it to international standards. The findings showed that utilizing GF with RAP in pavement construction has no environmental hazards, as the GF binder lowers heavy metal leaching in the RAP-FA mixture. Dayal and Nagan (2018) [32] explored the potential of employing GF as a coating for aggregates and its impact on an asphalt mixture’s properties. The GF was formed by using FA as an aluminosilicate source and NaOH and Na${}_{2}$SiO${}_{3}$ as alkaline activators. Aggregate testing, SEM analysis of FA and GF, Marshall flow and stability, indirect tension, and repetitive load tests on asphalt mixtures were performed during this study to investigate the influence of GF. The results revealed that the GF based on FA could be utilized to coat the natural aggregate and improve its physical and mechanical properties. Furthermore, using GF to coat aggregates in asphalt mixtures would improve the mixture’s stability and service life and could thus be used as a corrective method for FA disposal.

Moreover, geopolymer was used as a modifier for asphalt binder, as summarized in Table 2. Ibrahim et al. (2016) [33] investigated the influence of GF on the physical characteristics and storage stability of the asphalt binder (80/100). The GF additives were prepared in a dry state using FA (class F) and alkaline activators (NaOH and Na${}_{2}$SiO${}_{3}$). Different tests, including ductility, viscosity, and softening point tests, were performed to investigate the physical characteristics of unmodified and modified asphalt binders. The findings showed that as the GF concentration was increased from 3% to 9%, the ductility of the asphalt binder decreased from approximately 150 cm to 70 cm, indicating that the stiffness of the modified asphalt binder increased. In addition, the modified asphalt binders were stable at high storage temperatures. Tang et al. (2018) [34] investigated geopolymer uses in warm mixed asphalt. NaOH and Na${}_{2}$SiO${}_{3}$ were utilized at various concentrations to activate the aluminosilicate in slag, metakaolin, and silica fume. The findings revealed that geopolymer has excellent performance and numerous advantages, such as lowering the mixing temperature and minimizing the cost.

Recently, Hamid et al (2020) [38] investigated the possibility of using GF as a modifier for asphalt binder by using a new technique to produce the GF, whereby the GF was used as a dry substance. The findings were that GF significantly impacts the rheological properties of asphalt binder. They also found that there are no promising effects on the microstructure of the asphalt binder. Rosyidi et al. (2020) [35] investigated GF’s effect on the chemical characteristics of asphalt binder by utilizing Fourier transform infrared spectroscopy (FTIR). The GF was prepared using FA (class F) and an alkaline activator (NaOH and Na${}_{2}$SiO${}_{3}$). The results showed that adding GF had no significant influence on the FTIR spectra, showing that the asphalt binder’s functional group had not changed. This could be because of the small (less than 10%) addition of GF, which had no influence on the FTIR spectra geometry of the modified asphalt binder. Physically (but not chemically), the reaction that occurs throughout the modified asphalt binder process was observed. Aside from that, using GF as a modifier increased the asphalt binder’s bonding workability, and the high work cohesion value indicated that the cracking resistance of the asphalt binder was enhanced.

Furthermore, Rosyidi et al. (2020) [35] used the surface free energy (SFE) test to evaluate the impact of GF on the adhesion properties between an asphalt binder and two types of aggregate (limestone and granite). The findings demonstrated that except for the samples containing 5% GF-modified asphalt binder, which demonstrated a slightly lower adhesion in comparison with the control sample, the samples with GF-modified asphalt binder had higher adhesion than the samples without the modification. Tang et al. (2020) [36] prepared the GF in a dry state using FA (class F) and alkaline activators (NaOH and Na${}_{2}$SiO${}_{3}$). Two types of GF were used; uncalcined (G) and calcined (AG). The solvent precipitation and chromatographic column test methods were performed to investigate the impact of GF on the saturate, aromatic, resin, and asphaltene (SARA) components of asphalt binders. The findings revealed that adding GF to the asphalt binder had no substantial influence on the SARA components and properties of the asphalt binder. As a result, GF has a lot of possibilities for use as modifiers for asphalt binders.

### Research Significance and Objectives

According to the literature review, the geopolymer additive increased the asphalt binder’s workability and the work cohesion value, which revealed that the cracking resistance of the asphalt binder increased. In addition, the adhesion between the asphalt binder modified using geopolymer and the aggregate was enhanced, while the impact of the geopolymer content on the asphalt binder’s fatigue and low-temperature crack resistance has not yet been studied. Additionally, it is crucial to assess how the geopolymer-modified asphalt binder and mixture would perform under conditions of aging and climate change. On the other hand, SBS was broadly used in many countries, and it significantly affected the rheology and performance of asphalt binders. As a result, it may be possible to demonstrate the advantages of utilizing geopolymer as an asphalt modifier by comparing its positive effects with those of other modifiers, such as SBS. Therefore, this research intended to evaluate the influence of GF and its combination with the SBS polymer on the crack resistance of the asphalt binder by looking into the following:

- Investigating the influence of additives and temperature on the rheological and low and intermediate performance of asphalt binders;
- Investigating the additive, temperature, and frequency impacts on the dynamic modulus of an asphalt mixture;
- Evaluating the additives’ effects on the moisture damage resistance of an asphalt mixture;
- Evaluating the cracking resistance of an unmodified and modified asphalt mixture.

## 2. Experiment and Methods

### 2.1. Materials

#### 2.1.1. Preparation of Geopolymer Additives

**Figure 1:**
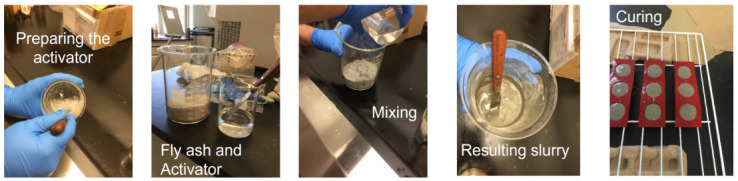
Geopolymer preparation.

In this study, GF was produced using an alkali activator and FA. The alkali activator consisted of (Na${}_{2}$SiO${}_{3}$) and NaOH at an 8 molar concentration. During the preparation of GF, the curing period and activator type were found to significantly influence the chemical reactions [39]. It is also worth mentioning that the combination of NaOH and Na${}_{2}$SiO${}_{3}$ gives geopolymer good mechanical performance [40]. Rifaai et al. (2019) [41] concluded that the highest storage modulus and yield stress values were achieved when 8 molar of NaOH is utilized as an activator in the production of GF. This study utilized low-calcium FA (Class F), which includes more silica and alumina and is therefore preferable to high-calcium FA (Class C), as many studies recommend [42,43,44]. The chemical composition of the fly ash is summarized in Table 3. The Na${}_{2}$SiO${}_{3}$ and NaOH solutions were used in a 1:2 mass ratio to activate the aluminosilicate precursors in the FA. The resulting slurry was then placed in silicon molds and cured for 6 days at 24 ± 1 °C and 1 day at 65 °C. After being ground into a powder, the GF samples were sieved through a No. 100 sieve. The procedure for producing the GF additives is depicted in Figure 1.

#### 2.1.2. Asphalt Binder Preparation

Two techniques were used in this investigation to blend the additives with the neat asphalt binder. In the first technique, the asphalt binder (PG 58-28) was heated to 140 °C before being blended with varying amounts of geopolymer (4%GF, 8%GF, and 12%GF) by utilizing a mechanical shear mixer at 2000 r/min for 60 min. Hamid et al. (2020) [38] noted that modifying asphalt binder using GF with different percentages (3, 6, and 9%) does not affect the asphalt binder’s microstructure. In another technique, the asphalt binder was heated to 170 °C ± 5 before being blended with 2% SBS by utilizing a high-shear mixer at 2000 r/min for 60 min. After that, the cross-linking agent was blended and stirred for 30 min at 10%. Lastly, curing time was carried out using 1000 r/min for 60 min at 180 °C ± 5. Table 4 presents the aggregate size distribution of the asphalt mixture. In order to investigate the effect of additives on the rheology and performance of the asphalt mixture, 5.3% of the total weight of the asphalt mixture was employed as a constant asphalt binder percentage in all asphalt mixtures.

#### 2.1.3. Aging Procedure

Following the mixing process of asphalt binders, samples for short-term aging in a rolling thin-film oven (RTFO) and long-term aging (20 h) in a pressure aging vessel (PAV) were prepared directly. The frequency sweep and LAS tests were performed using the DSR on all PAV samples. In addition, the low-temperature performance of the asphalt binders was investigated by performing the BBR test and measuring the creep stiffness (S) and relaxation rate (m) parameters.

### 2.2. Experimental Work

#### 2.2.1. Dynamic Shear Rheometer

The rheological behavior of the asphalt binders was evaluated utilizing the DSR according to AASHTO T 315 [45]. The influence of the loading frequency, temperature, and modification rate on the rheological characteristics of the asphalt binders was evaluated using a frequency sweep test. Each binder had two samples evaluated, and the test results were calculated using the average. For various test temperatures (5, 10, 15, 20, 25, and 30 °C), several frequencies varying from 0.0159 Hz to 15 Hz were used. A plate 8 mm in diameter with a 2 mm gap was used in a frequency sweep test to assess the effects of temperatures and the modifier content on the complex shear modulus (G${}^{*}$) and phase angle (δ). The DSR-LAS test was performed according to AASHTO TP 101 [46] for the neat and modified asphalt binders by utilizing a plate 8 mm in diameter and a 2 mm gap at different intermediate temperatures (10 °C, 20 °C, and 30 °C). Prior to the strain sweep test, a frequency sweep test was carried out to determine how the undamaged material responded. Subsequently, a constant frequency of 10 Hz was used in the strain sweep test to accelerate fatigue damage. In this study, the test findings were analyzed using the VECD models to predict the asphalt binder’s fatigue life (N${}_{f}$).

#### 2.2.2. Bending Beam Rheometer

The bending beam rheometer (BBR) test was performed in accordance with ASTM D6648 [47] to identify the flexural creep stiffness (S) and m value by means of a bending beam rheometer. Various temperatures (−12 °C, −18 °C, and −24 °C) were selected to investigate the effect of additives on the S and m value parameters of the asphalt binder.

#### 2.2.3. Dynamic or Complex Modulus Test

**Figure 2:**
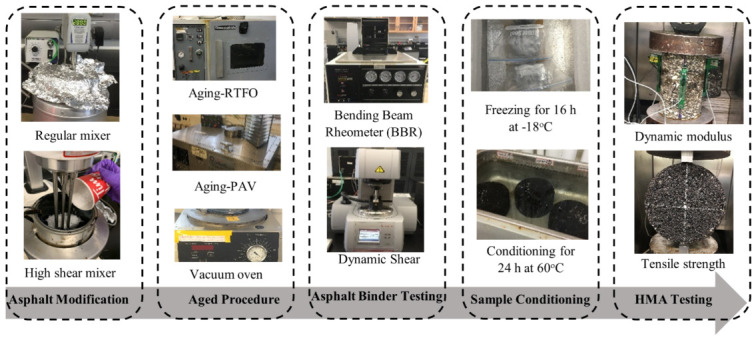
Experiment and methods.

The dynamic modulus test, as depicted in Figure 2, was performed to estimate the dynamic modulus (E${}^{*}$) and δ and to study the effects of different modifiers on the performance of the asphalt mixtures. The specimens were first prepared by heating the asphalt binders and aggregates in the oven to the appropriate mixing temperatures. The viscosities of the asphalt binders, using a rotational viscometer, were utilized to calculate the mixing and compaction temperatures of the asphalt mixtures. Prior to compaction, the asphalt mixtures conducted a 4 h conditioning period at 135 °C to reproduce the short-term aging of the asphalt mixes in accordance with AASHTO R 30 [48]. The specimens were then cored to the standard specimens, which were 100 mm in diameter and 150 mm in height. The CPATT lab’s MTS machine was used to test the cored specimens using different frequencies (0.1, 0.5, 1, 5, 10, and 25 Hz) and various temperatures (−10, 4, 21, 37, and 54 °C).

#### 2.2.4. Moisture Sensitivity Testing

The moisture damage evaluation was conducted in accordance with AASHTO T 283 [49]. Six specimens were prepared using the Superpave gyratory compactor, with dimensions of 150 mm in diameter and 95 mm in height and an air void content of 7 ± 0.5 %. These specimens were divided into conditioned and unconditioned subsets (each with three specimens) with almost the same average air void percentage. The conditioned subset was vacuumed to a saturation level between 70% and 80% before being frozen for 16 h at −18 °C and then thawed in a water bath for 24 h at 60 °C. Before testing, the two subsets were conditioned for 2 h at 25 °C. A 50 mm/min axial force was used during the strength testing to apply pressure until the peak load was reached. Equation (3) was then utilized to compute the indirect tensile strength ($S_{t}$):

(3) $$S_{t}=\frac{2000\times P}{\pi \times D\times t}$$

where $S_{t}$ is the tensile strength (kPa), *P* is the peak load (N) that the specimen can resist, *t* is the specimen thickness (mm), and *D* is the diameter of the specimen (mm).

## 3. Results and Discussion

### 3.1. Rheological Characteristics and Performance of Asphalt Binder

#### 3.1.1. Master Curve

**Figure 3:**
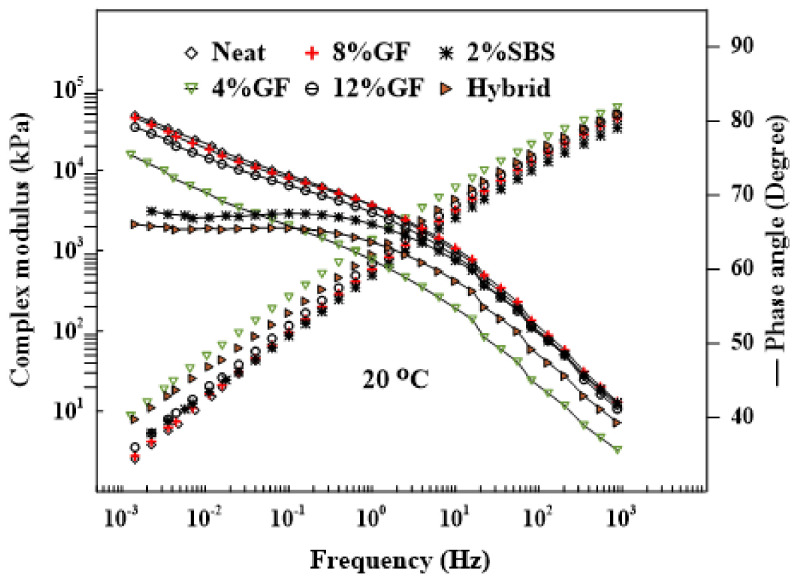
Master curve of asphalt binders at 20 °C.

Figure 3 displays the master curve for δ and G${}^{*}$ at 20 °C. The results indicated that there was a substantial effect from the modifiers on the viscoelastic behavior of the asphalt binders, where the additives increased the G${}^{*}$ and decreased the δ. However, it was impossible to discern which type of asphalt binder was more elastic or viscous based on the results for the phase angles. As a result, the complex shear moduli’s storage and loss modulus should be separated. Table 5 shows the characteristics of the aged (RTFO and PAV) and unaged asphalt binders.

#### 3.1.2. Temperature Susceptibility for the Loss and Storage Moduli

**Figure 4:**
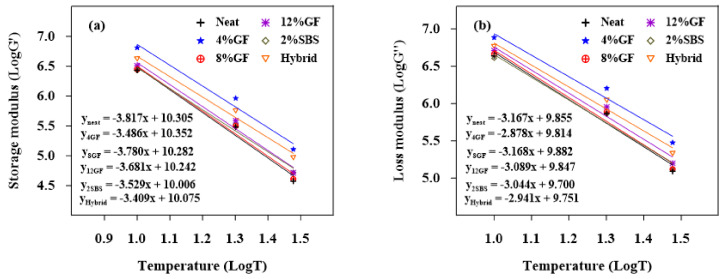
Sensitivity of unaged binders to temperature for (**a**) G′ and (**b**) G″.

**Figure 5:**
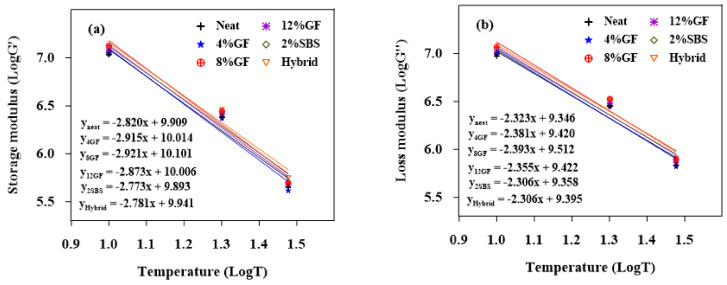
Sensitivity of PAV binders to temperature for (**a**) G′ and (**b**) G″.

**Figure 6:**
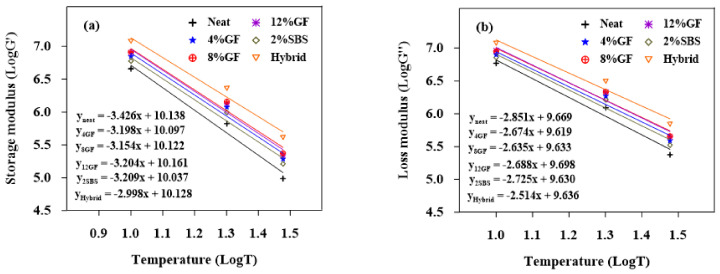
Sensitivity of RTFO binders to temperature for (**a**) G′ and (**b**) G″.

Figure 4, Figure 5 and Figure 6 show the logarithms of G′ and G″ vs. temperatures for the aged and unaged asphalt binders. The generated models for linear regression are shown in the figures. The absolute values of the slopes of the linear regression models are displayed in Table 6. The slope demonstrates the asphalt binders’ sensitivity to temperature. The sensitivity of a binder to temperature changes increased with the slope. The results show that the unaged and RTFO-modified asphalt binders had less temperature sensitivity for both G′ and G″. Low magnitudes for the G″ slope refer to lower susceptibility to the temperature. When the binders were exposed to long-term aging (PAV), the 8%GF binder became more susceptible to temperature changes.

#### 3.1.3. Fatigue Performance

**Figure 7:**
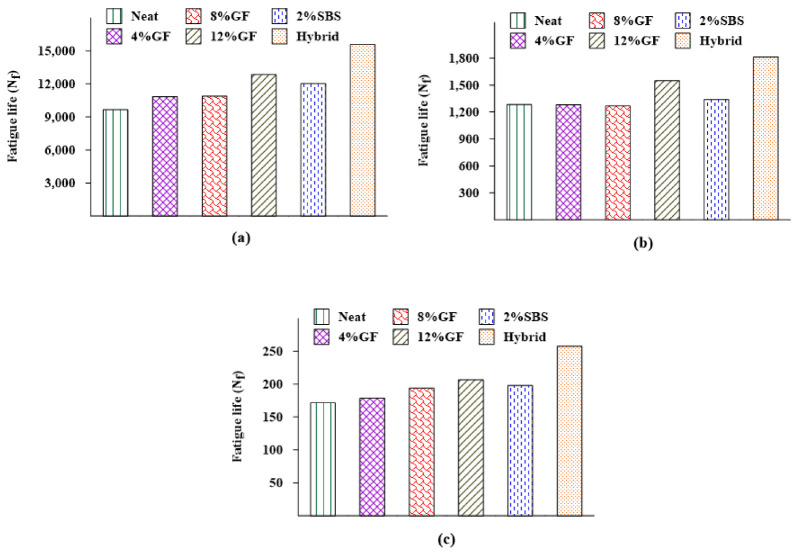
Additives effects on the N${}_{f}$ at (**a**) 10 °C, (**b**) 20 °C, and (**c**) 30 °C.

Figure 7 demonstrates the impact of modifiers on the N${}_{f}$ of asphalt binders at various temperatures (10 °C, 20 °C, and 30 °C). In this study, the N${}_{f}$ was calculated as the load repetitions at which the G${}^{*}$ decreased to 50% of its initial value. At various temperatures, it is obvious that the additives had a longer N${}_{f}$ than the neat binder. Meanwhile, the hybrid binder outperformed the other binders in terms of N${}_{f}$ at various temperatures. For example, the hybrid binder increased the N${}_{f}$ by 61%, 41%, and 50% at 10 °C, 20 °C, and 30 °C, respectively, compared with the neat asphalt binder. In contrast to the neat bin, 4%GF, 8%GF, and 2%SBS binders, the 12%GF binder had the maximum N${}_{f}$. For example, using 12%GF increased the N${}_{f}$ by 33%, 21%, and 20% at 10 °C, 20 °C, and 30 °C, respectively, compared with the neat binder. The findings indicate that the asphalt binder’s fatigue performance was significantly impacted by the GF modification, as was also concluded by Hamid et al. (2019) [9].

#### 3.1.4. Low Temperature Performance

**Figure 8:**
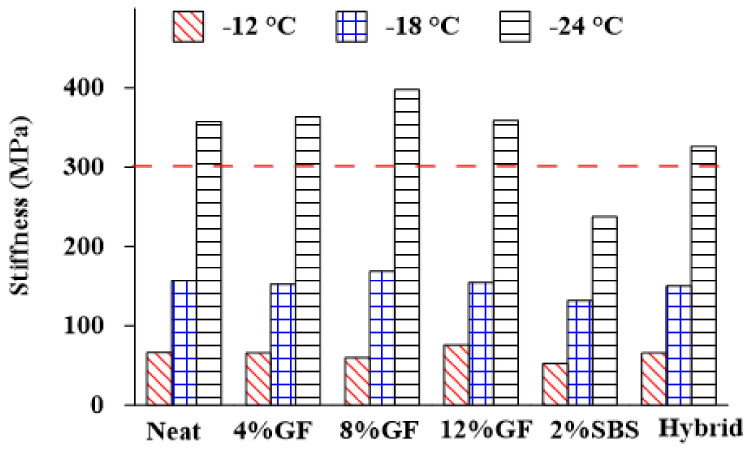
Effect of additives on the creep stiffness.

The flexural creep stiffness and rate of stress relaxation (m value) are parameters related to the low-temperature cracking resistance of asphalt binders. A higher value (>300 MPa) for the creep stiffness causes less resistance to thermal cracking. Likewise, a lower value (<0.3) for the m value induces a lower ability to absorb stress when the temperature drops and exhibits a higher cracking tendency [50]. Figure 8 represents the effect of additives and different temperatures (−12 °C, −18 °C, and −24 °C) on the creep stiffness of neat and modified asphalt binders. When the temperature was −12 °C, the creep stiffness of the GF-modified asphalt binder was reduced with additional GF by up to 8%, which indicated that the asphalt resistance to low-temperature cracking increased, while the 2%SBS mixture achieved the lowest creep stiffness compared with the neat binder and other modifiers.

There was a critical change in the behavior of the modified asphalt binder when the temperature was −18 °C and −24 °C. When the temperature was −18 °C, the creep stiffness of the 8%GF binder increased, indicating that the resistance of the asphalt binder to low-temperature cracking decreased. However, it met the condition of having a creep stiffness of less than 300 MPa. On the other hand, the 2%SBS binder achieved a creep stiffness of less than 300 MPa, which met the requirement that the creep stiffness be less than 300 MPa, whereas the other modifiers did not meet the requirement at −24 °C.

**Figure 9:**
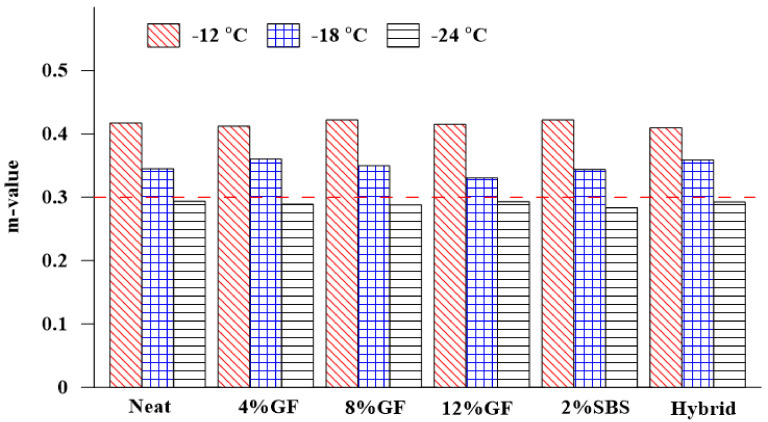
Effect of additives on the m value.

Figure 9 shows the influence of the additives and temperature on the rate of stress relaxation (m value). When the temperature was −12 °C, the 8%GF and 2%SBS binders attained the highest m values compared with other modifiers, which indicates that the stress relaxation performance increased. A higher m value refers to the binder’s ability to absorb stress in the case of a temperature drop and exhibit a lower cracking tendency [50]. Meanwhile, the 4% GF and hybrid binders exhibited more improvement in stress relaxation performance at −18 °C. The results indicate that all asphalt binders met the condition of having an m value of more than 0.3 at −12 °C and −18 °C, while at a temperature of −24 °C and 4% and 8% GF content, the m value change trend had a point of inflection, and the value was lower than the value of the base asphalt. The 12%GF and hybrid binders achieved approximately the same m value as the neat asphalt binder. None of the asphalt binders satisfied the requirement of having an m value more than 0.3 at −24 °C.

#### 3.1.5. Aging Effect

In this investigation, the crossover modulus was used to assess the impact of aging on the asphalt binders, which has been used to evaluate the relaxation properties [51] and oxygen uptake due to oxidation aging [52] in asphalt binders. Jing et al. (2020) [51] investigated the influences of the aging time, temperature, and pressure on the crossover modulus and concluded that aging has a significant impact on an asphalt binder as the crossover modulus reduces. Additionally, there is a better relationship between the inverse crossover modulus and the carbonyl changes in asphalt binders due to aging [52,53,54,55].

**Figure 10:**
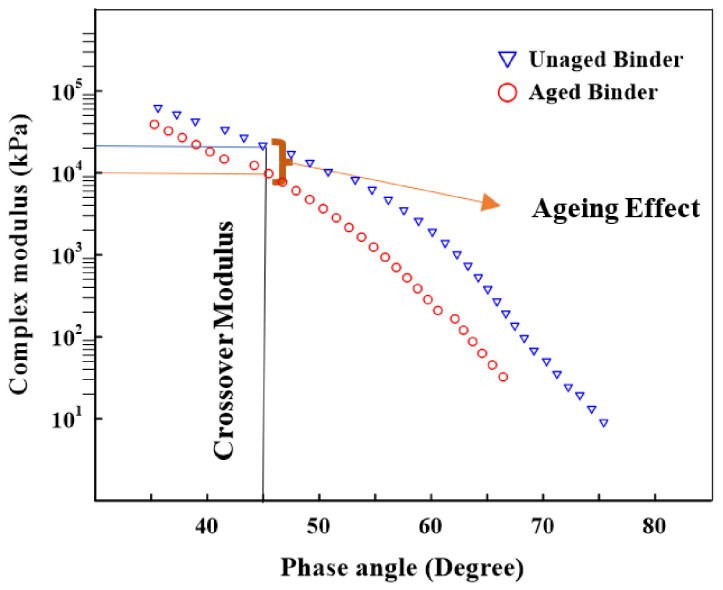
Evaluating the aging influence on the crossover modulus.

The crossover modulus (G${}_{c}$${}^{*}$) was calculated from the relationship between G${}^{*}$ and δ in the black diagram. The crossover modulus, shown in Figure 10, is related to a δ of 45°, where the storage modulus and loss modulus have the same value. The material acts more as a solid (elastic) when the δ is less than 45°. When it is more than 45°, the material behaves more as a fluid (viscous). If there are more data points in the region with a δ greater than 45°, then this shows that the asphalt binder is becoming more fluid (viscous reaction) and less solid (elastic response) as it ages. An aging index was proposed to measure the aging effect on an asphalt binder’s crossover modulus [51], which is represented as the ratio of the G${}_{c}$${}^{*}$ of an unaged asphalt binder over the G${}_{c}$${}^{*}$ of an aged asphalt binder:

(4) $$AI=\frac{G_{c-unaged}^{*}}{G_{c-aged}^{*}}$$

where AI is the aging index of the crossover modulus, $G_{c-unaged}^{*}$ is the crossover modulus of the unaged asphalt binder, and $G_{c-aged}^{*}$ is the crossover modulus of the unaged asphalt binder.

**Figure 11:**
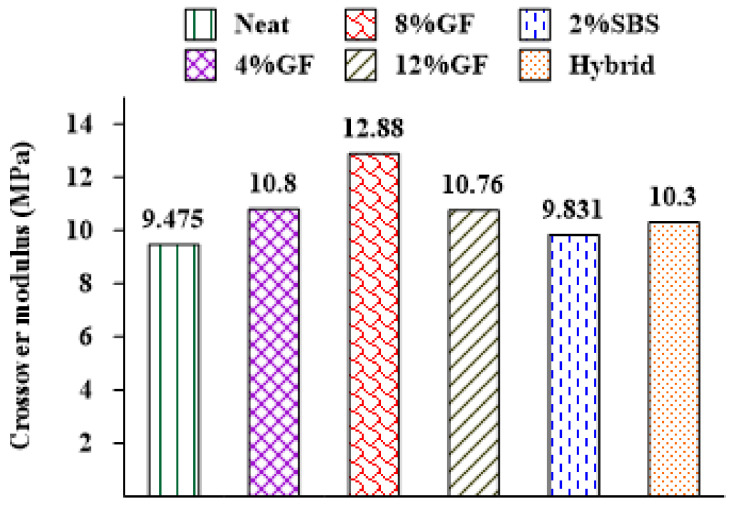
Additives’ influences on the crossover moduli of asphalt binders.

Figure 11 shows the crossover moduli of long-term aged neat and modified asphalt binders at 20 °C. The results demonstrate that the modified asphalt binder had the highest crossover modulus compared with the unmodified binder. This was ascribed to decreasing the influence of aging on the asphalt binder based on previous research [51,52].

**Figure 12:**
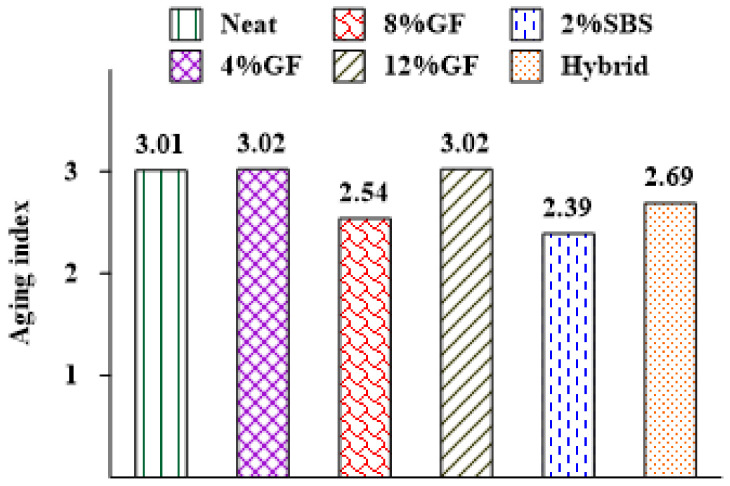
Additives’ influences on the aging indexes of asphalt binders.

The findings revealed that the modifiers had a considerable impact on the crossover modulus, where the crossover modulus of the neat asphalt binder was enhanced by adding modifiers. For example, the crossover modulus increased by 36% when 8%GF was added to the neat asphalt binder, demonstrating that the GF had a considerable impact on the viscoelastic behavior. In contrast, the 2%SBS binder exhibited low resistance to oxidation aging, as it achieved the smallest crossover modulus value. Figure 12 represents the change in the crossover modulus between the unaged and aged asphalt binders. The 8%GF and hybrid binders showed the same rate of change in the crossover modulus because of aging, while the smallest rate was obtained by adding 2% SBS.

### 3.2. Additives’ Effects on the Asphalt Mixture Performance

#### 3.2.1. Dynamic or Complex Modulus Test Analysis

The stress–strain relationship in an HMA is characterized by its dynamic modulus ($E^{*}$), which determines the stiffness properties of the HMA as a function of the temperature and loading rate under continuous sinusoidal loading [56]. The $E^{*}$ was considered a significant parameter input in the MEPDG design method for asphalt pavement, which is used for characterizing asphalt mixtures [57]. By conducting stress- or strain-controlled laboratory tests at several temperatures and frequencies, the $E^{*}$ of an asphalt mixture can be determined. It is possible to compare the stiffnesses of the asphalt mixtures over a variety of frequencies and temperatures by analyzing dynamic or complex modulus test data, which also includes the construction of the master curves.

**Figure 13:**
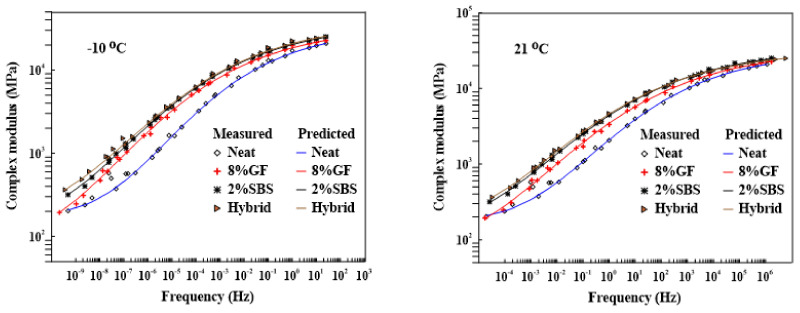
Dynamic or complex modulus master curves at −10 °C and 21 °C.

The linear viscoelastic characteristics of the asphalt binder and mixture at various frequencies and temperatures have been described by constructing the master curves using different models and shift factors. Rowe et al. (2009) [58] remarked that the generalized logistic sigmoidal model works well for the analyzed samples and suggested that it be used to obtain a better master curve for non-symmetric curves. Pellinen et al. (2002) [59] concluded that using the sigmoidal fitting function to generate the master curve for the $E^{*}$ test data appears to fit the data well, since it matches the physical shape of the measured data throughout a variety of temperatures. In this study, the shift factor ($a_{t}$) using the Williams, Landel, and Ferry (WLF) equation (Equation (5)) and the generalized logistic sigmoidal model (Equation (6)) were utilized to develop the master curve of an asphalt mixture with different temperatures (−10 °C and 21 °C), as depicted in Figure 13. The master curves were developed by utilizing a Microsoft Excel spreadsheet and the solver function. Table 7 summarizes the coefficients of the shift factor and sigmoidal model equations for various additives at different temperatures. The results revealed that the asphalt mixtures including various modified asphalt binders were stiffer than the asphalt mixtures containing the neat asphalt binder, which had the maximum $E^{*}$ at various frequencies:

(5) $$Log\;a_{t}=\frac{-C_{1}\left(T-T_{ref}\right)}{C_{2}+\left(T-T_{ref}\right)}$$

where $T_{ref}$ is the reference temperature and $C_{1}$ and $C_{2}$ are constants to reduce the difference between the actual and predicted data [60]:

(6) $$Log\;E^{*}\left(f,T\right)=\delta +\frac{\alpha }{\left(1+\lambda \exp^{(}\beta +\gamma \left(Logf_{r}\right)\right){)}^{\frac{1}{\lambda }}}$$

where *f* is the frequency, *T* is the temperature, δ is the lower asymptote, α is the difference between the values of the lower and upper asymptotes, β and γ are the shape coefficients, and λ is used to allow the curve to have a non-symmetrical shape [60].

**Figure 14:**
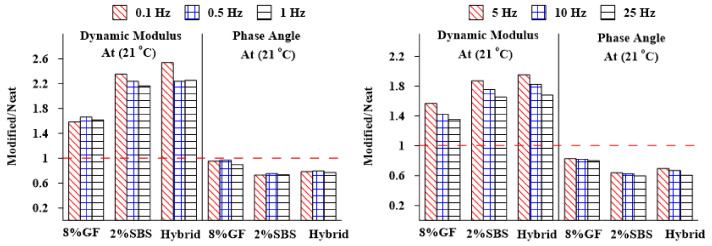
Frequencies’ effects on the $E^{*}$ and δ of different asphalt mixtures at 21 °C.

**Figure 15:**
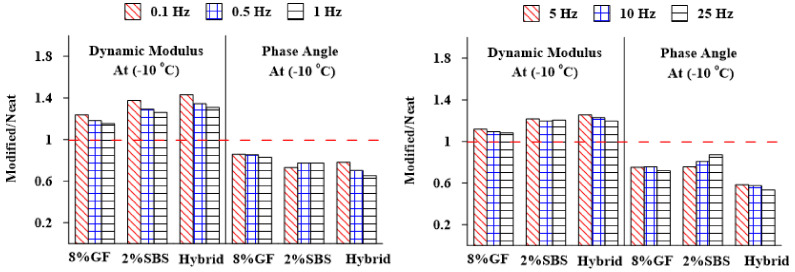
Frequencies’ effects on the $E^{*}$ and δ of different asphalt mixtures at −10 °C.

Figure 14 and Figure 15 show the $E^{*}$ and δ values measured for mixtures with various types of additives at various frequencies (0.1, 0.5, 1, 5, 10, and 25 Hz) and temperatures (−10 and 21 °C) but normalized to those obtained for the asphalt mixture with a neat binder. The findings show that the asphalt mixtures with modified binders had the maximum values of $E^{*}$ at low temperature (−10 °C and intermediate (21 °C) temperatures compared with the unmodified asphalt mixture, whereas there was a reduction in the δ at several frequencies and temperatures, which indicates that the viscoelastic behavior of the asphalt binder changed to be more elastic.

#### 3.2.2. Moisture Sensitivity Evaluation

Moisture damage in pavement materials is the most important issue that affects the durability of asphalt pavement. Moisture damage causes stripping, which is defined as a decrease in the adhesive bonding force between the binder and the aggregate particles. Moisture damage in asphalt mixes is caused by a loss of adhesion or cohesion, resulting in a progressive deterioration of the mixture’s strength and stiffness [50]. The tensile strength ratio (TSR), which compares the $S_{t}$ before and after conditioning, is used to determine how sensitive an asphalt mixture’s moisture content is.

**Figure 16:**
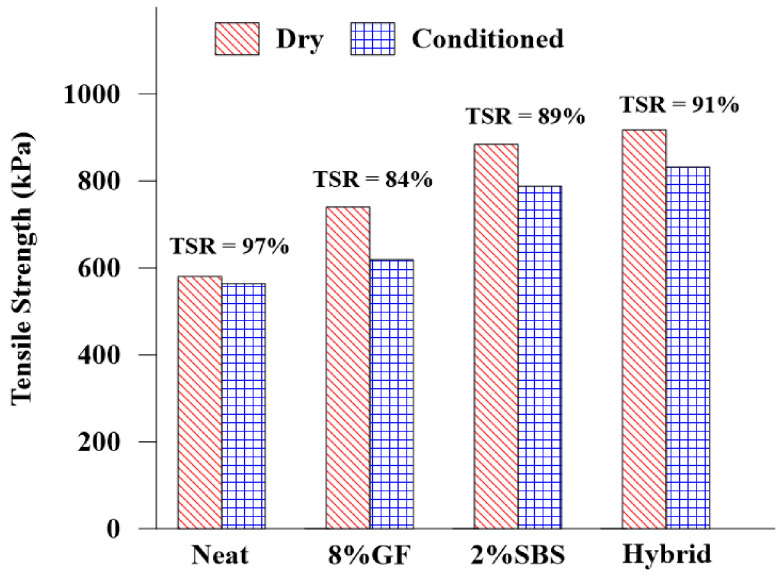
Moisture sensitivity evaluation.

Figure 16 demonstrates the impacts of different modifiers on the $S_{t}$ of the asphalt mixture before and after conditioning. In the dry condition, the $S_{t}$ increased from 580.4 kPa to 740.4 kPa, 884.8 kPa, and 917.4 kPa by using 8%GF, 2%SBS, and hybrid binders, respectively. In the freezing-thawing condition, there was a high reduction in the $S_{t}$ by using 8%GF, 2%SBS, and hybrid binders, but the $S_{t}$ still increased from 563.7 kPa to 618.9 kPa, 787.8 kPa, and 832.1 kPa, respectively.

The findings suggest that both the neat and modified asphalt binder mixes were sensitive to moisture damage, as the asphalt mixtures achieved tensile strength ratio (TSR) values of more than 80%. The asphalt mixtures with different modifiers (8%GF, 2%SBS, and hybrid) had higher $S_{t}$ values than the neat asphalt binder, which was attributed to the modifications increasing the adhesion between the asphalt binder and aggregate. It was observed that the hybrid mix was the least susceptible to moisture damage, with a TSR of 91% compared with the other modifiers. According to the findings by Rosyidi et al. (2020) [35] as well, this outcome demonstrated that the asphalt binder modified with GF was significantly more resistant to moisture damage.

#### 3.2.3. Asphalt Mixture Fracture Energy

**Figure 17:**
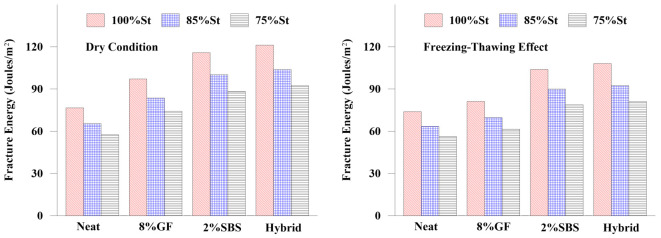
Influence of additives on the asphalt mix’s fracture energy.

Additives’ effects on the macro cracking resistance of asphalt mixes and the impact of freezing and thawing on the cracking resistance of asphalt mixes were investigated in Figure 17. One of the factors that determines an asphalt mix’s cracking resistance is fracture energy. Asphalt mixes with higher fracture energy have improved crack resistance [61]. After the peak stress, the load-bearing capacity of the asphalt mix will certainly decline as a macro crack begins to form and grows. Therefore, to evaluate the influences of additives on the cracking resistance of asphalt mixes, the fracture energy was measured at the peak $S_{t}$ and at the dropping of the $S_{t}$ to 85% and 75% using Equations (7) and (8), respectively:

(7) $$W_{f}=\sum_{i=1}^{n-1}\left(\left(l_{i+1}-l_{i}\right)\times P_{i}+\frac{1}{2}\left(l_{i+1}-l_{i}\right)\times \left(P_{i+1}-P_{i}\right)\right)$$

(8) $$G_{f}=\frac{W_{f}}{Dt}\times 10^{6}$$

where $G_{f}$ is the failure energy (Joules/m${}^{2}$), $W_{f}$ is the failure work (Joules), *D* is the diameter (mm) of the specimen, and *t* is the thickness of the specimen (mm). The results revealed that the use of GF, SBS, and the mixture of GF and SBS increased the asphalt mixture’s cracking resistance. The hybrid binder attained the highest fracture energy in dry and freezing-thawing conditions, indicating the most effective cracking resistance among the modifiers. It was also indicated that the freezing-thawing condition had a negative impact on the fracture energy of modified asphalt mixtures due to the presence of water, which caused a reduction in the adhesion between the asphalt binder and aggregate. Despite this reduction, the modified asphalt mixes still had higher fracture energies than the mixtures made with a neat asphalt binder.

## 4. Conclusions

In this study, the rheological and performance characteristics of asphalt binders modified with GF and SBS polymer were investigated. The influences of additives, temperatures, and aging on asphalt binder performance were also evaluated. Meanwhile, the influence of additives on moisture damage and cracking resistance were investigated. The results could lead to the following conclusions:

- The asphalt binder modified with 12%GF attained the highest N${}_{f}$ compared with the neat, 4% GF, 8%GF, and 2%SBS binders. Compared with the neat asphalt binder, the N${}_{f}$ increased by 33%, 21%, and 20% at 10 °C, 20 °C, and 30 °C, respectively. Compared with the neat asphalt binder, the hybrid modified asphalt binder increased the N${}_{f}$ by 61%, 41%, and 50% at 10 °C, 20 °C, and 30 °C, respectively.
- The thermal cracking performance of the asphalt binder was improved when the 2%SBS and hybrid modifiers were used, achieving the lowest creep stiffness.
- Using 8% GF improved the neat asphalt binder’s crossover modulus. Compared with the neat asphalt binder, adding 8% GF increased the crossover modulus by 36%, indicating a significant effect of GF on the viscoelastic behavior, whereas adding 2% SBS and hybrid modifiers to the asphalt binder increased the crossover modulus by 4% and 9%, respectively.
- The asphalt mixes with different modifiers had higher $S_{t}$ values than the neat asphalt binder. When using the 8% GF, 2% SBS, and hybrid binders, the $S_{t}$ increased from 580.4 kPa to 740.4 kPa, 884.8 kPa, and 917.4 kPa, respectively. The asphalt mixture with a hybrid binder had the least susceptibility to moisture damage, with a TSR of 91% in comparison with other modified asphalt binders. This finding indicates that combining SBS and GF as a modifier for asphalt binders demonstrated a significant level of moisture resistance.
- Due to the presence of water, which reduces the adhesion between the asphalt binder and aggregate, the fracture energies of the modified asphalt mixes were negatively impacted by the freeze-thaw condition. Despite this reduction, the modified asphalt mixes still had higher fracture energies than the mixtures prepared with a neat asphalt binder.

The findings of this investigation demonstrate that the application of geopolymer significantly affected the rheology and performance of the asphalt binders. Only fly ash was used to produce the geopolymer, and an eight-molar solution of Na${}_{2}$SiO${}_{3}$ and NaOH served as the alkali activator. Preparing geopolymer from various aluminosilicate sources, such as slag, clay, glass powder, or a combination of these, will be more interesting. It would be more productive to verify the efficiency of the geopolymer and asphalt binder using various alkaline activator types at various concentrations, such as 4, 6, 8, 10, and 12 molar. Additionally, combining SBS and GF had a substantial effect on the asphalt binder’s resistance to aging, fatigue, and thermal cracking. Therefore, the investigation of various GF/SBS ratios’ effects on the rheology and performance properties is in our plan for future research. Furthermore, the performance of asphalt mixtures modified with GF and the combination of GF and SBS has also received minimal attention. This research is a key point from which to start comprehensive laboratory work to investigate the fatigue and thermal cracking resistance of a modified asphalt mixture, considering the impact of temperatures, frequencies, aging, and the freezing-thawing condition.

## Figures and Tables

**Table 1:** Using geopolymers as an effective stabilizer for soil.

Reference	Soil Type	Geopolymer		Main Comments
		Activator	Pozzolanic	
[22]	Clay	NaOH+ Na${}_{2}$SiO${}_{3}$	MK	For clay soils, metakaolin-geopolymer can be employed as a soil stabilizer.
[23]	Silty sand	NaOH+ Na${}_{2}$SiO${}_{3}$	FA+S+RG	Improves the durability and strength of the soil.
[24]	Silty clay	NaOH+ Na${}_{2}$SiO${}_{3}$	FA	Improves the compressive strength of the soil.
[25]	Sandy clay	NaOH+ Na${}_{2}$SiO${}_{3}$	FA	Strength significantly changes as the ratio of activator to ash is reduced.
[26]	Silty clay	Na${}_{2}$SiO${}_{3}$+ CCR+ water	FA	CCR can be used as an alternative alkaline activator to produce GF.
[27]	Silt, sand, and clay	NaOH+ Na${}_{2}$SiO${}_{3}$	FA+ CCR	GF enhances the strength and is considered an effective green soil stabilizer.
[28]	Clay	Na${}_{2}$SiO${}_{3}$+ CCR	S	Improves water absorption, permeability, and strength.
[19]	Clay	NaOH	FA+S	Improves the compressive strength of the soil.
[29]	Silt, sand, clay, and gravel	NaOH (or KOH)+ Na${}_{2}$SiO${}_{3}$	FA	Increasing FA/soil ratio causes a considerable increase in compressive strength.

Note: MK = metakaolin; FA = ﬂy ash; S = slag; RG = red gypsum; CCR = calcium carbide residue.

**Table 2:** Using geopolymer as a modifier for asphalt binder.

Reference	Asphalt Type	Additive State	Geopolymer		Main Comments
			Activator	Pozzolanic	
[33]	80/100	Gel	NaOH+ Na${}_{2}$SiO${}_{3}$	FA (F)	Asphalt binder modified with GF could remain stable at high storage temperature.
[34]	AH-90	Dry	NaOH+ Na${}_{2}$SiO${}_{3}$	MK+S+SF	Geopolymer was suitable additive for developing high-performing WMA.
[35]	80/100	Gel	NaOH+ Na${}_{2}$SiO${}_{3}$	FA (F)	Asphalt modified with GF had improved storage stability and structural chain mobility characteristics.
[36]	AH-90	Dry	NaOH+ Na${}_{2}$SiO${}_{3}$	MK+S+SF	The potential of geopolymer to absorb bitumen VOCs and PM emissions was relatively high.
[9]	PG58-28	Dry	NaOH+ Na${}_{2}$SiO${}_{3}$	FA+GP	The characteristics of the asphalt binder could be significantly enhanced as a result of geopolymer application.
[37]	PG58-28	Dry	NaOH+ Na${}_{2}$SiO${}_{3}$	FA	Enhancing rutting performance using GF as asphalt binder modifiers was proven to be effective.

Note: MK = metakaolin; FA = ﬂy ash; S = slag; SF = silica fume; GP = glass powder; VOCs = volatile organic compounds.

**Table 3:** Chemical composition of fly ash [37].

Constituent (%)	SiO_2_	Al_2_O_3_	Fe_2_O_3_	CaO	MgO	SO_3_	Na_2_O	MC	LOI
Fly ash	57.2%	23.5%	3.8%	9.3%	1.0%	0.2%	2.43%	0.06%	0.77%

Note: MC = moisture content; LOI = loss on ignition.

**Table 4:** Size distribution of aggregate [37].

Sieve Size (mm)	Passing (%)	Control Point (Maximum)	Control Point (Minimum)
19	100		
12.5	95	100	90
9.5	83	90	28
4.75	58		
2.36	40	58	28
1.18	19		
0.6	12		
0.3	8		
0.15	4.5		
0.075	3	10	2

**Table 5:** Asphalt binder properties.

Parameter	Neat	2%SBS	Hybrid	4%GF	8%GF	12%GF
**Orginal Asphalt Binder**						
Rotational Viscosity @ 135 °C	0.417	0.678	0.900	0.531	0.661	0.561
Rotational Viscosity @ 165 °C	0.119	0.191	0.235	0.146	0.179	0.160
G${}^{*}$/sin δ ( kPa) @ 52 °C, at 1.6 Hz	5.036	6.756	14.532	7.517	9.606	7.244
**RTFO-Asphalt Binder**						
G${}^{*}$/sin δ ( kPa) @ 52 °C, at 1.6 Hz	9.73	14.68	36.71	16.82	21.52	17.81
% Recovery @ 52 °C, at 3.2 kPa	8.72	45.14	48.97	18.83	24.67	19.28
J${}_{nr}$ @ 52 °C at 3.2 kPa	0.84	0.27	0.09	0.39	0.27	0.38
**PAV-Asphalt Binder**						
Stiffness Critical Temperature (T${}_{s}$)	−32.7	−35.7	−33.4	−32.7	−32.0	−32.7
Slope Critical Temperature (T${}_{m}$)	−33.2	−31.5	−33.3	−33.1	−32.8	−32.9
G${}^{*}$/sin δ ( kPa) @ 20 °C, at 1.6 Hz	2817	3006	3304	2829	3360	3069

**Table 6:** Regression slope of unaged, RTFO, and PAV binders.

Binders	The Slope $|G^{\prime }|$			The Slope $|G^{\prime \prime }|$		
	Unaged	RTFO	PAV	Unaged	RTFO	PAV
Neat	3.817	3.426	2.820	3.167	2.851	2.323
2%SBS	3.529	3.209	2.773	3.044	2.725	2.306
Hybrid	3.409	2.998	2.781	2.941	2.514	2.306
4%GF	3.486	3.198	2.915	2.878	2.674	2.381
8%GF	3.780	3.154	2.921	3.168	2.635	2.393
12%GF	3.681	3.204	2.873	3.089	2.688	2.355

**Table 7:** Summary of the shift factor and sigmoidal model coefficients.

Temperature	Binder	Shifting Coefficients		Model Coefficients					
		**C** ${}_{1}$	**C** ${}_{2}$	α	β	δ	γ	λ	**R** ${}^{2}$
−10 °C	Neat	35.029	205.401	2.244	−1.953	2.257	−0.372	0.125	0.999
	8%GF	31.837	170.798	2.569	−2.225	1.960	−0.311	0.064	0.999
	2%SBS	29.515	163.013	2.389	−2.181	2.173	−0.312	0.046	0.999
	Hybrid	33.829	192.746	2.299	−2.096	2.284	−0.306	0.042	0.998
21 °C	Neat	28.484	220.324	2.234	−0.250	2.256	−0.377	0.131	0.999
	8%GF	27.124	205.103	2.589	−0.717	1.939	−0.314	0.065	0.999
	2%SBS	23.141	179.736	2.355	−0.725	2.184	−0.324	0.047	0.999
	Hybrid	17.179	129.899	2.256	−0.742	2.253	−0.329	0.042	0.999

## Data Availability

All data used during the study appear in the submitted article.
